# Evaluation of different agricultural wastes for the production of polysaccharides from *Oudemansiella raphanipes* and its antioxidant properties

**DOI:** 10.1002/fsn3.2945

**Published:** 2022-07-08

**Authors:** Qi Wei, Xinrong Zhong, Maryam Hajia Haruna, Shengrong Liu, Fengfang Zhou, Meixia Chen

**Affiliations:** ^1^ College of Life Science Ningde Normal University Ningde China; ^2^ Industry and University Research Cooperation Demonstration Base in Fujian Province Ningde China; ^3^ Fujian Higher Education Center for Local Biological Resources in Ningde Ningde China; ^4^ Engineering Research Center of Mingdong Aquatic Product Deep‐Processing Fujian Province University Ningde China; ^5^ National Animal Production Research Institute Ahmadu Bello University Zaria Nigeria

**Keywords:** agricultural wastes, antioxidant activity, *Oudemansiella raphanipes*, polysaccharides

## Abstract

*Oudemansiella raphanipes* (OR) is a commercial mushroom which possesses high nutritional value and excellent and unique flavors. In this study, various agricultural wastes were utilized as substitute materials in the low‐cost and high‐yield production of mycelia biomass and polysaccharides by liquid fermentation. The sawdust, wheat bran, apple pomace, sugarcane, and corn particles were employed to cultivate OR, using the potato dextrose broth as control. Additionally, a preliminary characterization and in vitro antioxidant activities of partial purified OR polysaccharides were investigated. The substrate of sugarcane was suitable for mycelia growth of OR, with high yield of mycelia biomass and polysaccharides content. In vitro antioxidant activity assays demonstrated that OR polysaccharides could effectively scavenge 2,2′‐azino‐*bis*(3‐ethylbenzothiazoline‐6‐sulfonic acid) and 1,1‐diphenyl‐2‐picrylhydrazyl radicals. OR polysaccharides had configuration as revealed by Fourier transform infrared, and was mainly composed of fucose (Fuc), rhamnose (Rha), arabinose (Ara), galactose (Gal), glucose (Glc), xylose (Xyl), mannose (Man), ribose (Rib), and galacturonic acid (Gal‐UA), with mass percentages of 3.29%, 0.64%, 1.09%, 16.03%, 72.69%, 0.56%, 3.18%, 0.93%, and 1.59%, respectively. This study may offer support for decreasing the cost of OR polysaccharides production and dealing with these agricultural wastes.

## Graphical abstract

The potential of selected agricultural wastes for the polysaccharides production of *Oudemansiella raphanipes* was assessed. The preliminary characterization and antioxidant activities of partial purified polysaccharides were investigated. Utilization of agricultural wastes in polysaccharides production is a profitable approach to waste management.

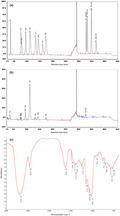

## 1 INTRODUCTION

The agricultural sector contributes significantly to global waste production. Examples include peels, cotton seed hulls, and coffee pulp generated from juice, cotton, and coffee processing, respectively. Consequently, improper management of these wastes could cause environmental pollution (Mishra and Satapathy, 2021; Kani et al., 2021). Interestingly, attributes possessed by these wastes, especially the nutritive value, attracts their utilization in directions with great economic and social benefits. The agricultural wastes have been considered as good source of nutrients for mushroom production because they support mycelia growth and development into mushroom fruit bodies. The most commercially used agricultural wastes for edible mushroom production include wheat bran, cotton seed hulls, sawdust, sugarcane bagasse, corn cobs, hard wood chips, rice and wheat straws (Chiu et al., 2000; Sadh et al., 2018). Several researches demonstrated the use of bahiagrass, banana stalks, and coffee husks as substrates for the cultivation of oyster mushroom (Siqueira et al., 2011; Murthy and Manonmani, 2008). Kumhomkul and Panich‐pat (2013) showed that the use of paddy straw as substrate was utilized for the production of straw mushroom. More so, agricultural wastes were beneficial for king oyster mushroom production, and they appeared to be the main ingredients responsible for its growth, development, and delayed nutrient release (Jeznabadi et al., 2016). It is an interesting approach to use agricultural wastes as a bioconservation for the cultivation of edible mushroom in a controlled way. Therefore, the mushroom cultivation is the most efficient and economically viable biotechnology to solve the environment‐related problems using agricultural wastes as a raw material.

*Oudemansiella raphanipes* (OR), commercially called “Heipijizong,” is an important edible mushroom in China. OR is considered an excellent delicacy and widely cultivated in many regions of China (Hao et al., 2016). OR possesses many nutritional and medicinal values, excellent and unique flavors, and high protein content. Moreover, research has identified the potential bioactivity of OR with antioxidant, antitumor, immunomodulatory, and hepatoprotection. The orcinol from OR showed antioxidant capacity via scavenging the radicals of DPPH (Zhou et al., 2020). The petroleum ether extract of OR showed enhanced anticancer properties (Guo et al., 2021). The polysaccharides are one of the most important bioactive compounds in OR. The immunomodulatory and antitumor ability of OR was suggested by Kim et al. (2007) to be related to its polysaccharides. It also has been reported that the polysaccharides of OR demonstrated antioxidant and hepatoprotective activities (Liu et al., 2017). Compared to extracting polysaccharides from OR fruit bodies, extracting the polysaccharides from OR mycelia using liquid fermentation is an effective, healthy, and environmental‐friendly method to obtain the polysaccharides of OR with high yield and low production costs. Therefore, the polysaccharides of OR are a potential resource for healthy food with high consumer demand. However, limited studies systematically investigated the potential use of agricultural wastes in the production of OR by liquid fermentation.

In this study, five different agricultural wastes, namely sawdust, wheat bran, apple pomace, sugarcane, corn powder, were evaluated for their suitability for the cultivation of OR by liquid fermentation. The biomass and polysaccharides produced by OR were compared among the five agricultural wastes, using potato dextrose broth medium as control.

## 2 MATERIALS AND METHODS

### 2.1 Materials and chemicals

2,2′‐Azino‐*bis*(3‐ethylbenzoth‐iazoline‐6‐sulfonic acid) (ABTS), 2,2‐diphenyl‐1‐picrylhydrazyl (DPPH), trichloroacetic acid, and Trolox were purchased from Sigma‐Aldrich (St Louis, MO), hydrogen peroxide (H_2_O_2_), potassium persulfate, and ferrous sulfate were purchased from Sinopharm Chemical Reagent Co., Ltd. and other analytical grade laboratory chemicals were utilized in this study.

### 2.2 Media preparation

The sawdust, wheat bran, apple pomace, sugarcane, and corn particles were dried at 55°C to a constant weight, crushed using a Chinese medicine crusher (2500Y; Xuman), and then filtered through 80‐mesh screen (SUS304, Olodo). Potato dextrose agar (PDA) contained 20 g/L glucose, 6 g/L potato infusion, and 20 g/L agar. Potato dextrose broth (PDB) contained 20 g/L glucose and 6 g/L potato infusion. Sawdust medium contained 15 g/L sawdust powder and 30 g/L glucose. Wheat bran medium contained 15 g/L wheat bran powder and 30 g/L glucose. Apple pomace medium contained 15 g/L apple pomace powder and 30 g/L glucose. Sugarcane medium contained 15 g/L sugarcane powder and 30 g/L glucose. Corn medium contained 15 g/L corn powder and 30 g/L glucose.

### 2.3 Fungal strain and liquid spawn preparation

A commercial strain of OR was deposited at the Mycological Research Center of Fujian Agricultural and Forestry University, Fuzhou, China, under the number OR‐0‐0001. The OR was used throughout this study. The OR was inoculated into PDA plates and incubated at 25°C for 7 days. A 250‐ml flask containing 100 ml of different liquid medium was inoculated with three pieces (5 mm in diameter) of mycelia plugs from PDA and incubated at 25°C with shaking of 150 rpm. To optimize the liquid cultivation of OR, the inoculated liquid medium was incubated with different shaking speed (100, 120, 150, 180, and 200 rpm), incubation temperatures (15, 20, 25, 30, and 35°C), and incubation duration (3, 5, 7, 10, and 12 days).

### 2.4 Estimation of mycelia biomass

After incubation, the mycelia of OR were harvested by filtration with three layers of cloth and then washed three times using distilled water. The harvested mycelia of OR were transferred into Petri dishes and dried at 55°C to a constant weight.

### 2.5 Extraction and purification of polysaccharides from OR

The polysaccharides of OR was determined according to the method described by Chen et al. (2018). The mycelia powder of OR was mixed in distilled water (1:30 w/v) and extracted by incubating in a water bath (HWS‐26, Shanghai Yiheng Technical, Co. Ltd.) at 80°C for 3 h. After extraction, the solvents were filtered through a Buchner funnel and centrifuged at 4500 rpm for 20 min to separate the pellets. The supernatant was mixed with trichloroacetic acid to remove the free protein before mixing with a fourfold volume of ethanol and kept at 4°C for 12 h. The particle purified polysaccharides of OR were harvested afterward using a refrigerated centrifuge (4°C, Sigma‐Aldrich Co. Ltd., MO) at 4500 rpm for 15 min and then freeze‐dried (FDU‐2110, Eyela).

### 2.6 Antioxidant activities in vitro

#### 2.6.1 DPPH radical scavenging activity

The DPPH radical scavenging activity has been widely used to evaluate the antioxidant potentials of antioxidants (Liao et al., 2012). The DPPH (8 mg) was dissolved in ethanol (45 ml) for 5 min using ultrasonic bath (KQ‐5000DE, Kunshan Ultrasonic Instrument, Co. Ltd.). Afterward, the ethanol was slowly added to the DPPH solution to reach an absorbance value of 1.2 at 517 nm using Varioskan LUX Microplate reader (Thermo Fisher Scientific, Co. Ltd.). The polysaccharides obtained from OR was dissolved in distilled water at different concentrations (0.5, 1, 2, 4, and 8 mg/ml). A volume of 100 μl of each concentration was then mixed with 100 μl of DPPH solution, vortexed immediately, and incubated in the dark for 30 min at room temperature before taking the absorbance reading at 517 nm using Trolox (Sigma‐Aldrich Co. Ltd., MO) as a control. The DPPH radical scavenging activity was calculated using the following equation:

(1) $$\text{DPPH scavenging activity}\;\left(\%\right)=\left[1-\frac{A_{i}-A_{j}}{A_{0}}\right]\times 100\%$$

where *A*_0_ is the absorbance of the blank (ethanol without any sample), *A*_i_ is the absorbance of the sample, and *A*_j_ is the absorbance of the control (ethanol without DPPH).

#### 2.6.2 ABTS assay

The ABTS radical scavenging activity was determined according to the method described previously with slight modification (Wei et al., 2020). In brief, ABTS (7 mM) and potassium persulfate (2.45 mM) solutions were mixed and incubated in the dark at 25°C for 12 h. After incubation, the mixed solution was diluted with distilled water to reach an absorbance value of 0.7 ± 0.05 at 734 nm. Next, 100 μl of the mixture was mixed with 100 μl of polysaccharides of OR sample, followed by incubation at room temperature for 6 min. The absorbance was measured at 734 nm using Trolox (Sigma‐Aldrich Co. Ltd., MO) as a control. The ABTS radical scavenging activity was measured using the following equation:

(2) $$\text{ABTS scavenging activity}\;\left(\%\right)=\left[1-\frac{A_{i}-A_{j}}{A_{0}}\right]\times 100\%$$

where *A*_0_ is the absorbance of the blank (distilled water without any sample), *A*_i_ is the absorbance of the sample, and *A*_j_ is the absorbance of the control (distilled water without ABTS).

### 2.7 Preliminary characterization of polysaccharides from OR

#### 2.7.1 Monosaccharide composition analysis

The monosaccharide composition of the polysaccharides of OR was evaluated by high‐performance anion‐exchange chromatography (HPAEC, ICS5000, Thermo Fisher Scientific, Co. Ltd.) equipped with a CarboPac PA‐20 anion‐exchange column (150 mm × 3.0 mm × 10 μm), with an injection volume of 5 μl. The binary gradient elution system consisted of (A) water (0.1 M sodium hydroxide) and (B) acetonitrile (0.1 M sodium hydroxide, 0.1 M sodium acetate). The separation was achieved using the following: 95–80% A over 0–30 min, 60% A over 30–45 min, and 95% A over 45–60 min at a flow rate of 0.5 ml/min. Identification of monosaccharide components was analyzed by comparison with standard monosaccharides of fucose (Fuc), rhamnose (Rha), arabinose (Ara), galactose (Gal), glucose (Glc), xylose (Xyl), mannose (Man), fructose (Fru), ribose (Rib), galacturonic acid (Gal‐UA), glucuronic acid (Glc‐UA), mannuronic acid (Man‐UA), and guluronic acid (Gul‐UA).

#### 2.7.2 Fourier transform infrared (FT‐IR) spectroscopy analysis

The FT‐IR spectrum analysis was recorded on a Fourier transform‐infrared spectrophotometer (Nicolet 6700, Thermo Fisher Scientific, Co. Ltd.) within the scope of 4000–500 cm^−1^, using the potassium bromide disc method to prepare the specimen.

### 2.8 Statistical analysis

The software SPSS 18.0 was used for data analysis and expressed as means ± standard deviation of three replicates. One‐way ANOVA was used for all statistical comparisons among groups, followed by mean comparisons using the Duncan's multiple‐range test at *p* < .05.

## 3 RESULTS

### 3.1 Polysaccharides and mycelia biomass production on different substrates

**FIGURE 1:**
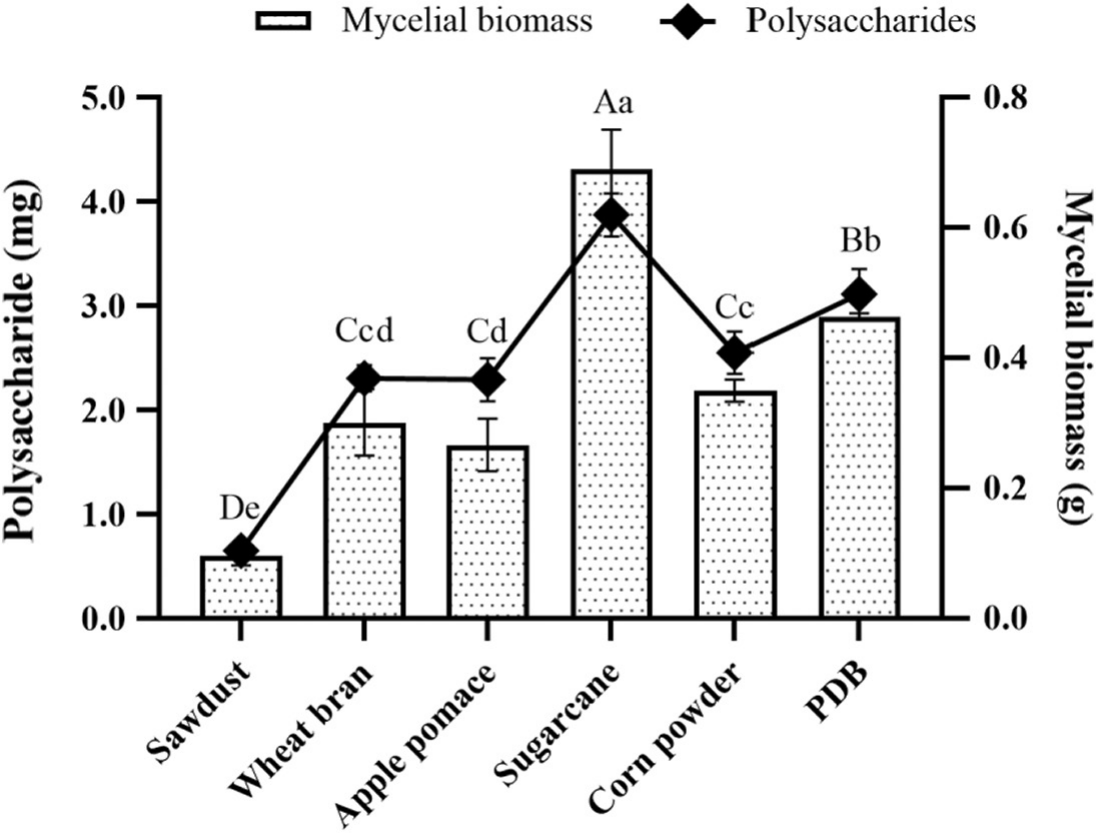
Effects of different substrates on the production of mycelia biomass and polysaccharides from the submerged mycelia culture of *Oudemansiella raphanipes* mushroom. Values marked by different letters are significantly different (*p* < .05)

The effects of different agricultural wastes on the polysaccharides and mycelia biomass production of OR were assessed in this study. Mycelia growth showed different growth rate on different agricultural wastes by liquid fermentation. The polysaccharides and mycelia biomass production of OR was significantly (*p* < .05) influenced by the substrate compositions. Sugarcane substrate had the highest polysaccharides (0.69 ± 0.05 g) and mycelia biomass (3.87 ± 0.18 mg) production of OR as compared to the other five liquid media assessed (Figure 1). PDB showed the second highest polysaccharides (0.46 ± 0.01 g) and mycelia biomass (3.11 ± 0.22 mg) production, while sawdust substrate showed the lowest polysaccharides (0.10 ± 0.01 g) and mycelia biomass (0.65 ± 0.11 mg) production. Hence, the substrate of sugarcane was used for subsequent investigations based on the revealed production of polysaccharides and mycelia biomass and viability in mycelia cultivation of OR.

### 3.2 Effects of rotation speeds on mycelia biomass and polysaccharides

**FIGURE 2:**
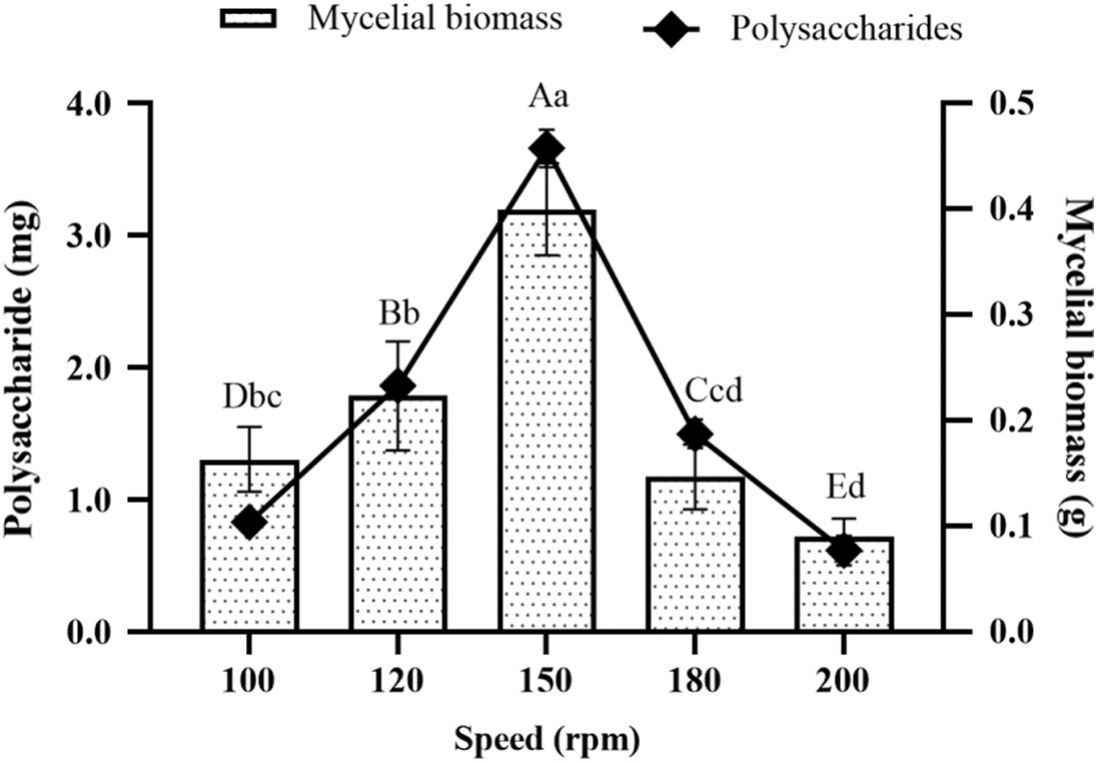
Effects of different rotation speeds on the production of mycelia biomass and polysaccharides from the submerged mycelia culture of *Oudemansiella raphanipes* mushroom. Values marked by different letters are significantly different (*p* < .05)

There are significant variations among different rotation speeds on the production of mycelia biomass (*p* < .05, Figure 2). The production of mycelia biomass varied from 0.09 ± 0.02 g to 0.40 ± 0.04 g for the different rotation speeds (100, 120, 150, 180, and 200 rpm). The increase in rotation speed from 100 rpm to 150 rpm consequently caused a rapid increase in the production of mycelia biomass and polysaccharides. Moreover, the peak rotation speed on OR growth which yielded the highest production of mycelia biomass (0.40 ± 0.04 g) and polysaccharides (3.66 ± 0.12 mg) was attained at 150 rpm. Afterward, a significant decline in the production of mycelia biomass and polysaccharides (*p* < .05) was observed at 180 rpm and 200 rpm compared to the rotation speed of 150 rpm. Thus, rotation speed of 150 rpm was considered as an appropriate rotation speed.

### 3.3 Effects of cultivation temperature on mycelia biomass and polysaccharides

**FIGURE 3:**
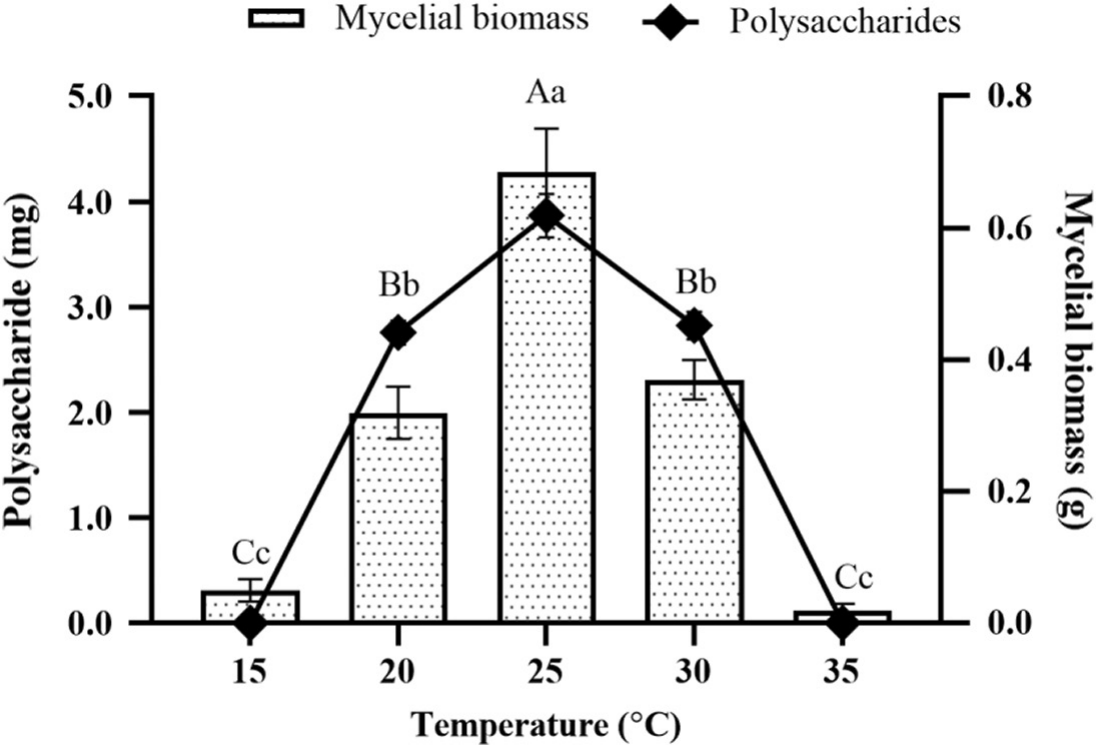
Effects of different cultivation temperatures on the production of mycelia biomass and polysaccharides from the submerged mycelia culture of *Oudemansiella raphanipes* mushroom. Values marked by different letters are significantly different (*p* < .05)

As shown in Figure 3, increasing temperature from 15°C to 25°C prompted a significant increase (*p* < .05) in the production of mycelia biomass and polysaccharides. Subsequent cultivation temperature increase (30°C and 35°C) revealed significant decrease (*p* < .05) in the production of mycelia biomass and polysaccharides. Accordingly, the cultivation temperature of 25°C which revealed the highest production of mycelia biomass (0.69 ± 0.06 g) and polysaccharides (3.87 ± 0.18 mg) is the most suitable.

### 3.4 Effects of cultivation duration on mycelia biomass and polysaccharides

**FIGURE 4:**
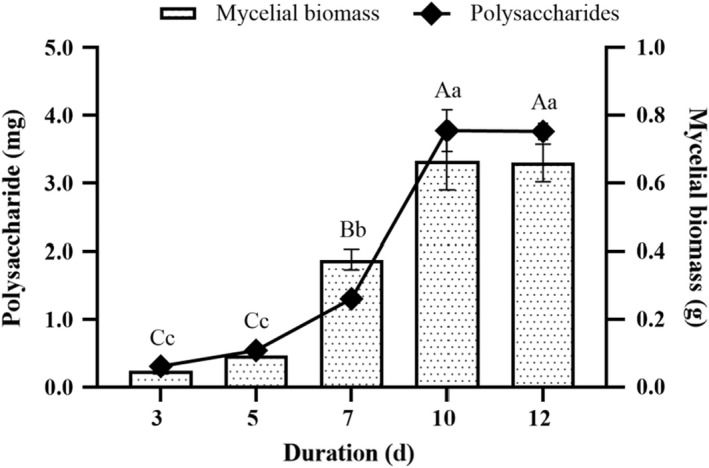
Effects of different cultivation duration on the production of mycelia biomass and polysaccharides from the submerged mycelia culture of *Oudemansiella raphanipes* mushroom. Values marked by different letters are significantly different (*p* < .05)

As shown in Figure 4, the production of mycelia biomass and polysaccharides increased with longer cultivation duration and peaked at 10 days, but the difference was insignificant after 10 days. Therefore, the cultivation duration of 10 days was considered as the suitable cultivation duration for mycelia growth with highest polysaccharides production by liquid fermentation.

### 3.5 Antioxidant activities of OR polysaccharides

**FIGURE 5:**
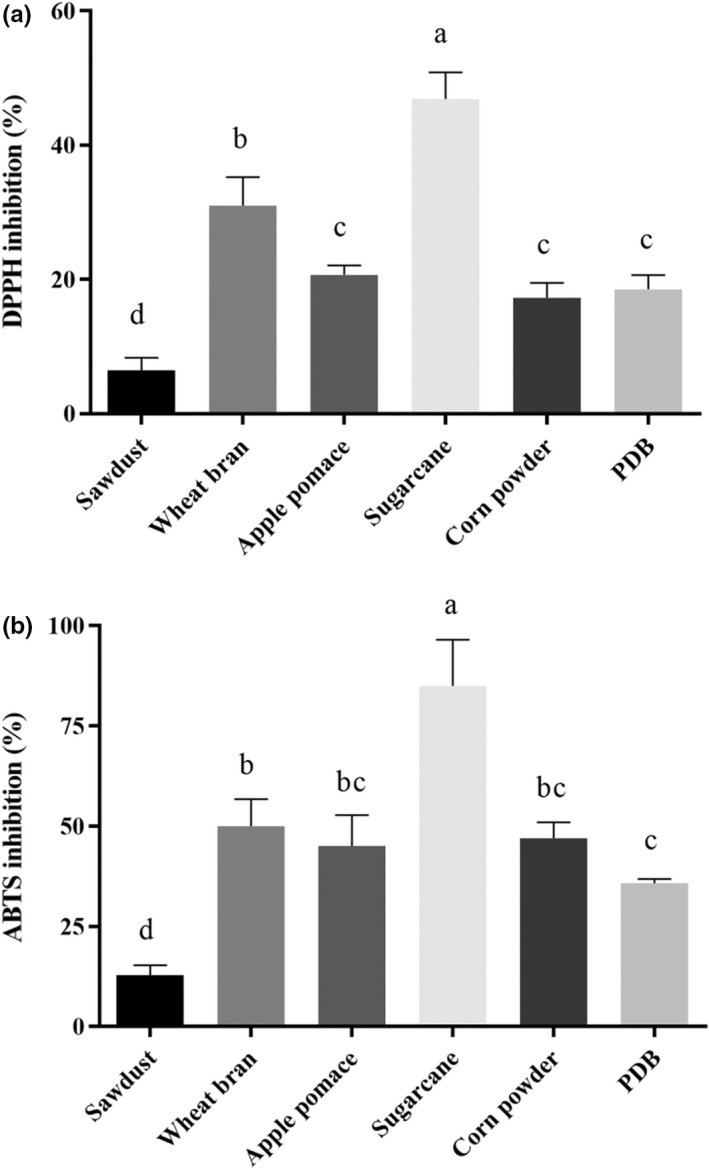
Antioxidant activities of polysaccharides from *Oudemansiella raphanipes* cultivated at different substrates. (a) DPPH inhibition and (b) ABTS inhibition. Values marked by different letters are significantly different (*p* < .05)

The antioxidant activities of OR polysaccharides extracted from different agricultural wastes were investigated by assessing the scavenging radicals activities of DPPH and ABTS (Figure 5). As shown in Figure 5, the polysaccharides extracted from sugarcane substrate showed the strongest DPPH (46.90 ± 3.53%) and ABTS (84.97 ± 10.33%) radicals scavenging activities, followed by wheat bran substrate. The values exhibited by the DPPH and ABTS radicals scavenging activity of polysaccharides extracted from apple pomace, corn powder, and PDB were not significantly different (*p* > .05). The polysaccharides extracted from sawdust substrate had the lowest DPPH (6.44 ± 1.70%) and ABTS (12.77 ± 2.25%) radicals scavenging activities.

**FIGURE 6:**
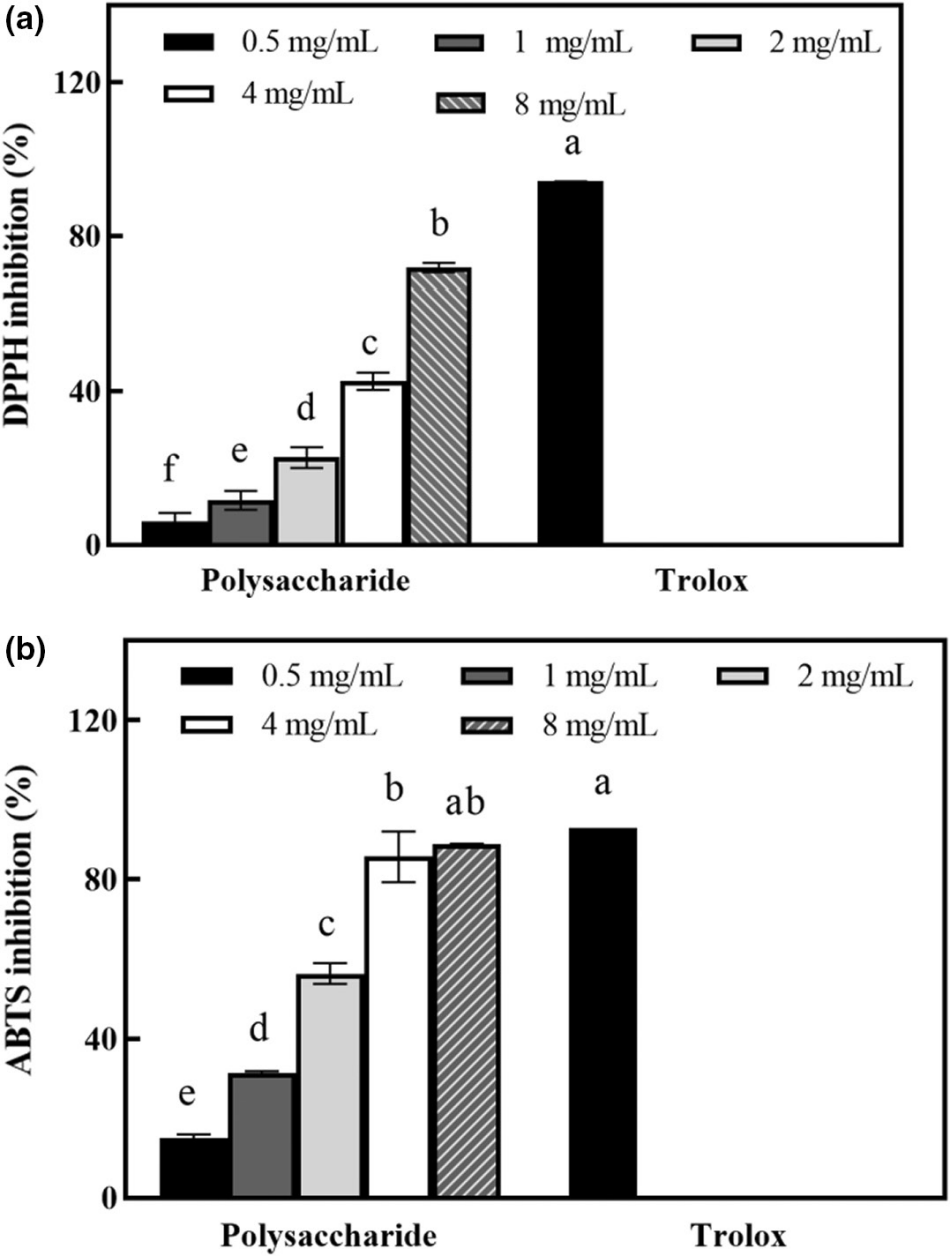
Antioxidant activities of polysaccharides from *Oudemansiella raphanipes*: (a) DPPH inhibition and (b) ABTS inhibition. Values marked by different letters are significantly different (*p* < .05)

As shown in Figure 6, the OR polysaccharides showed increasing scavenging activities on DPPH and ABTS radicals at higher concentrations. At the concentration of 0.5 mg/ml, the DPPH radicals scavenging activity of OR polysaccharides was 6.21 ± 1.83%. At concentration of 8 mg/ml, the DPPH radicals scavenging activity of OR polysaccharides (71.80 ± 1.09%) was significantly higher than that of 0.5, 1, 2, and 4 mg/ml (*p* < .05). The concentration of OR polysaccharides to inhibit 50% (IC_50_) of DPPH was 5.28 mg/ml (Figure 6a). The ABTS scavenging ability of OR polysaccharides was measured at different concentrations, ranging from 0.5 to 8 mg/ml. At the concentration of 0.5 mg/ml, the ABTS radicals scavenging activity of OR polysaccharides was 15.09 ± 0.73%. As the concentrations of OR polysaccharides were increased, the ABTS radical scavenging activity also increased. The OR polysaccharides showed an outstanding scavenging activity with an ABTS inhibition value of 88.75 ± 0.24% at 8 mg/ml concentration. The IC_50_ of OR polysaccharides was 2.51 mg/ml (Figure 6b).

### 3.6 Characterization analysis of OR polysaccharides

**FIGURE 7:**
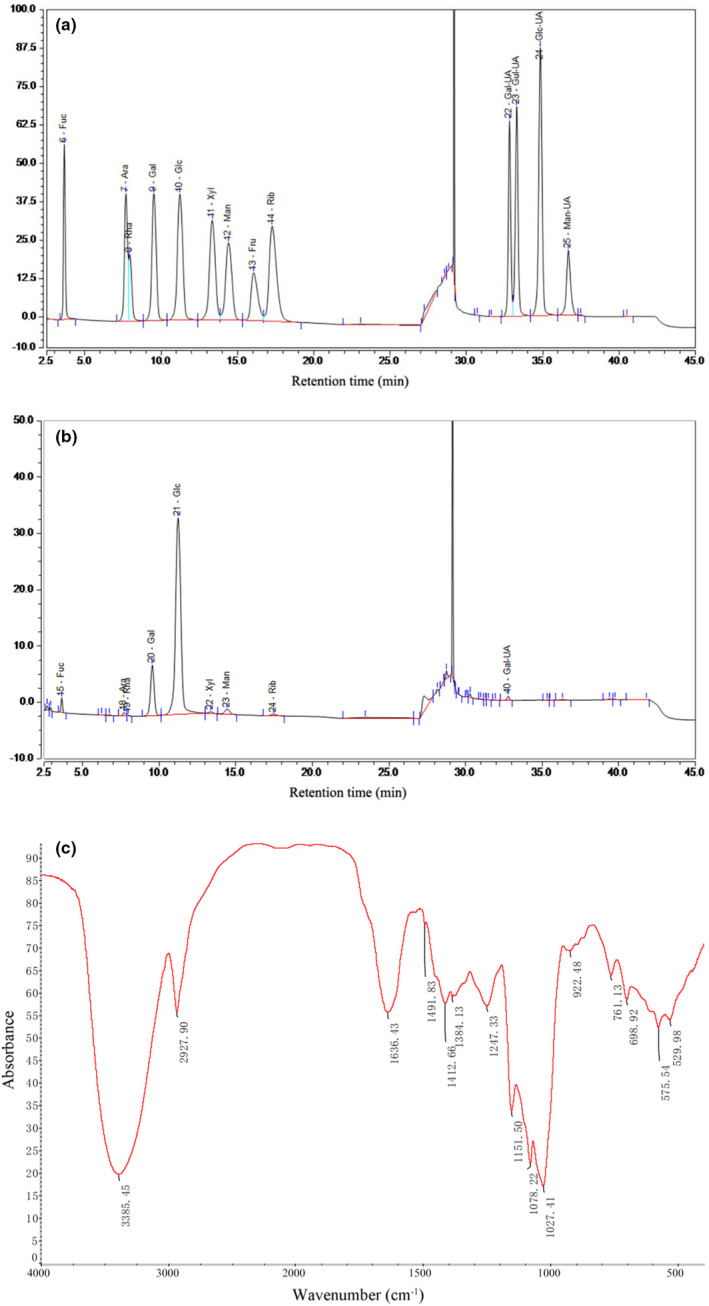
Preliminary characterization of polysaccharides from *Oudemansiella raphanipes*. (a) HPAEC chromatograms of standard monosaccharides, (b) HPAEC chromatograms of OR polysaccharides, and (c) FT‐IR spectrum

The monosaccharide composition of the polysaccharides of OR was investigated by comparison with the retention time of the standard samples' chromatographic peaks including Fuc, Rha, Ara, Gal, Glc, Xyl, Man, Fru, Rib, Gal‐UA, Glc‐UA, Man‐UA, and Gul‐UA (Figure 7a). The polysaccharides of OR were made up of nine different monosaccharides of Fuc, Rha, Ara, Gal, Glc, Xyl, Man, Rib, and Gal‐UA with mass percentages of 3.29%, 0.64%, 1.09%, 16.03%, 72.69%, 0.56%, 3.18%, 0.93%, and 1.59%, respectively (Figure 7b). Thus, Glc was the major monosaccharide composition of the polysaccharides of OR.

The FT‐IR spectra is a rapid technique useful in polymer structure and functional groups identification. FT‐IR is capable of qualitative identification of the polysaccharides structure. Thus, FT‐IR was employed in the characterization analysis of OR polysaccharides. A wide and strong band at 3385.45 cm^−1^ representing OH stretching vibrations, peaks at 2927.90 cm^−1^, 1636.43 cm^−1^, at range of 1450 cm^−1^ to 1200 cm^−1^, 1151.50 cm^−1^, 1078.22 cm^−1^ and 1027.41 cm^−1^ representing ‐C‐H stretching vibrations, C = O stretching vibrations, combined with C‐H stretching vibrations attributed to characteristic absorption of sugar rings, C‐O‐C glycosidic bonds vibrations, and C‐O bending vibrations, respectively, were displayed in Figure 7c. The diagnostic absorption peaks at 922.48 cm^−1^ may suggest the presence of β‐d‐pyranoid glucose. The results suggest that the sample had typical structure of polysaccharides, specifically a pyranose form sugar with β configurations.

## 4 DISCUSSION

The vast amount of the agricultural wastes were produced by agricultural‐based industries every year. Most of the agricultural wastes are untreated and underutilized. The agricultural wastes without proper disposal procedure that may cause to environmental pollution. Mushroom production is a noticeable method of reducing environmental pollution by using agricultural wastes as a raw material. The cultivation of mushrooms which requires carefully controlled biological system creates an ecological as well as economical means for the valorization of agricultural wastes, thereby promoting bioconservation. The essentiality of agricultural wastes as substrates in mushroom production owing to their nutritive value have been reported by researchers (Graminha et al., 2008). Rezaeian and Pourianfar (2016) reported that different agricultural wastes including sugarcane, soybean meal, wheat straw, wheat bran, and sawdust could be used for the cultivation of *Flammulina velutipes*. In this study, the agricultural wastes such as sawdust, wheat bran, apple pomace, sugarcane, and corn particles were used for the production of mycelia biomass and polysaccharides from OR. The substrate of sugarcane exhibited greater suitability to the mycelia growth of OR by liquid fermentation. This is probably due to the relatively higher content of sucrose in sugarcane (Lin et al., 2016). Therefore, the substrate of sugarcane was selected for the liquid fermentation. The growth of mycelia was affected by various factors, especially the rotation speed, cultivation temperature, and duration. In this study, the optimal cultivation conditions for the mycelia growth of OR were at 25°C in orbital shaker (150 rpm) for 10 days. Under the optimal conditions, the mycelia of OR would grow well, and increase the production of mycelia biomass. Thus, it is an effective and economical method to produce the OR polysaccharides on a large scale.

The DPPH is a stable radical which can be destroyed in the presence of antioxidants. The DPPH radical scavenging activity has been applied to the measurement of the radical scavenging abilities of antioxidants. The ABTS radicals can be reduced in the presence of such hydrogen‐donating antioxidants. The ABTS assay is applicable to the study of both water‐soluble and lipid‐soluble antioxidants (Re et al., 1999). The DPPH and ABTS assays have been widely used for describing the antioxidant abilities of antioxidants (Liao et al., 2012; Chen and Kang, 2014). The natural antioxidants without any side effects could provide an effective treatment through counteracting the oxidative stress related to an excess of reactive oxygen species (Teng and Chen, 2017). Polysaccharides from mushroom having medicinal properties could offer an attractive strategy to manage oxidative stress and as functional food for human. The antioxidant activities of OR polysaccharides were investigated by assessing the scavenging activity of free radicals. The OR polysaccharides showed an excellent ability to scavenge DPPH and ABTS radicals (Figures 5 and 6).

The polysaccharides of *Agaricus blazei* Murrill extracted by compound enzymes (cellulase:pectinase:papain) exhibited antioxidant abilities via scavenging values of ABTS radicals, DPPH radicals, and hydroxyl radicals (Jia et al., 2016). The polysaccharides of *Oudemansiella radicata* exhibited the ability to scavenge hydroxyl, ABTS, and DPPH radicals, and chelate ferrous ion. The DPPH and ABTS radical scavenging activity of polysaccharides from *O. radicata* with an IC_50_ value of 0.9 mg/ml and 2.3 mg/ml, respectively, was reported by Wang et al. (2018). In this study, the DPPH radical scavenging activity of OR polysaccharides (IC_50_ 5.28 mg/ml) was not as strong as that of polysaccharides from *O. radicata* (IC_50_ 0.9 mg/ml). However, the ABTS radical scavenging activity of OR polysaccharides (IC_50_ 2.51 mg/ml) was similar to that of polysaccharides from *O. radicata* (IC_50_ 2.3 mg/ml) reported by Wang et al. (2018). Therefore, the polysaccharides of OR effectively scavenged the radicals of DPPH and ABTS, suggesting its potential use as antioxidant agents.

A HPAEC analysis of OR polysaccharides showed nine different monosaccharides and a dominance of Glc, followed by Gal, Fuc, Man, Gal‐UA, Ara, Rib, Rha, and Xyl. The monosaccharides composition of OR polysaccharides were similar to that of alkali‐extracted *Coprinus comatus* fruiting body polysaccharides which includes Glc, Gal, Fuc, Man, Rib, Rha, and Xyl (Zhao, Lai, et al., 2018). Moreover, OR polysaccharides had the β configuration as revealed by FT‐IR spectrum analysis, which is in accordance to the report of Dore et al. (2014). Also, the polysaccharides configuration from *Polyporus albicans* is composed of β configuration. Song et al. (2018) demonstrated that fucose plays a valid role in conferring biological activities, and the β‐type glycosidic linkages contribute to the biological activities. The OR polysaccharides exhibited the antioxidant activities, which may be related to the β‐type glycosidic linkages. However, polysaccharides configuration from *Cordyceps militaris* having α and β configurations, and *Grifola frondosa* having α configuration were different from the configuration of OR polysaccharides (Meng et al., 2016; Zhao, Zhang, et al., 2018), which could be as a result of difference in the strains. Putting together, the funding of this study supports the exploration of agricultural wastes in the production of OR polysaccharides, thereby decreasing the production cost to a greater extent.

## 5 CONCLUSIONS

In this study, the potentials of different substrates on the production of mycelia biomass and polysaccharides from OR were investigated. The substrate of sugarcane was beneficial for the mycelia growth, with highest production of mycelia biomass and polysaccharides. To investigate the additional information on the function of OR polysaccharides, their physicochemical characteristics and in vitro antioxidant activities were studied. The OR polysaccharides showed the ability to scavenge DPPH and ABTS radicals in vitro. Furthermore, HPAEC analysis showed that the OR polysaccharides comprised nine kinds of monosaccharides, including Fuc, Rha, Ara, Gal, Glc, Xyl, Man, Rib, and Gal‐UA. FT‐IR analysis showed that OR polysaccharides possesses the typical absorption features of polysaccharides with β‐configuration pyranose. Ultimately, this study booted the idea that utilization of agricultural wastes for the production of polysaccharides by liquid fermentation can be beneficial in solving the environmental problems as well as providing natural nontoxic and functional foods for humans.

## CONFLICT OF INTEREST

The authors declare that they do not have any conflict of interest.

## Data Availability

The data that support the findings of this study are available from the corresponding author upon reasonable request.
